# Human-aided admixture may fuel ecosystem transformation during biological invasions: theoretical and experimental evidence

**DOI:** 10.1002/ece3.966

**Published:** 2014-02-23

**Authors:** Jane Molofsky, Stephen R Keller, Sébastien Lavergne, Matthew A Kaproth, Maarten B Eppinga

**Affiliations:** 1 Department of Plant Biology, University of VermontBurlington, Vermont, 05405; 2 Appalachian Laboratory, University of Maryland Center for Environmental ScienceFrostburg, Maryland, 21532; 3 Laboratoire d'Ecologie Alpine (LECA) UMR 5553 CNRS - Université Joseph Fourier BP 53Grenoble Cedex 9, 38041, France; 4 Department of Ecology, Evolution & Behavior, University of MinnesotaSaint Paul, Minnesota, 55108; 5 Department of Environmental Science, Copernicus Institute of Sustainable Development, Utrecht UniversityUtrecht, TC 3508, The Netherlands

**Keywords:** Admixture, critical transitions, dynamics, ecosystems, feedbacks, functional traits, invasive species, *Phalaris arundinacea*, thresholds

## Abstract

Biological invasions can transform our understanding of how the interplay of historical isolation and contemporary (human-aided) dispersal affects the structure of intraspecific diversity in functional traits, and in turn, how changes in functional traits affect other scales of biological organization such as communities and ecosystems. Because biological invasions frequently involve the admixture of previously isolated lineages as a result of human-aided dispersal, studies of invasive populations can reveal how admixture results in novel genotypes and shifts in functional trait variation within populations. Further, because invasive species can be ecosystem engineers within invaded ecosystems, admixture-induced shifts in the functional traits of invaders can affect the composition of native biodiversity and alter the flow of resources through the system. Thus, invasions represent promising yet under-investigated examples of how the effects of short-term evolutionary changes can cascade across biological scales of diversity. Here, we propose a conceptual framework that admixture between divergent source populations during biological invasions can reorganize the genetic variation underlying key functional traits, leading to shifts in the mean and variance of functional traits within invasive populations. Changes in the mean or variance of key traits can initiate new ecological feedback mechanisms that result in a critical transition from a native ecosystem to a novel invasive ecosystem. We illustrate the application of this framework with reference to a well-studied plant model system in invasion biology and show how a combination of quantitative genetic experiments, functional trait studies, whole ecosystem field studies and modeling can be used to explore the dynamics predicted to trigger these critical transitions.

## Introduction

It is increasingly clear that interactions between ecological and evolutionary processes have the potential to affect biodiversity at multiple scales of organization (Whitham et al. 2006; Bailey et al. 2009; Wymore et al.^2011^), leading to the long anticipated integration of evolutionary biology and ecosystem science (Matthews et al. 2011). This conceptual unification of function and diversity across scales, from intraspecific variation in traits to community stability and ecosystem function, has the potential to transform our understanding of how evolutionary changes organize and structure ecological systems (Matthews et al. 2011; Schoener 2011). In particular, there is mounting evidence that genetic changes within a foundation or dominant species (sensu Ellison et al. 2005) can cause a cascade of changes in associated communities of interacting species (Hanski 2011; Schoener 2011). For example, genetic changes in a foundation plant species can alter community diversity of co-existing herbivores because different herbivore communities develop on different host plant phenotypes (Bailey et al. 2009; Keith et al. 2010; Genung et al. 2011; Lamit et al. 2011; Schweitzer et al. 2011).

At the same time, there has been a growing realization that changes in functional traits of dominant plant species can induce a critical transition toward a novel ecosystem state (Suding et al. 2008). Critical transitions are typically preceded by a gradual loss of resilience of the current ecosystem state, making the system vulnerable to relatively small perturbations or (further) changes in environmental conditions or pressures (Scheffer et al. 2001). Hence, critical transitions might appear as large-scale ecosystem responses to what are arguably smaller-scale changes in functional variation within a dominant species (Eppinga et al. 2011). However, much is still unknown about how micro-evolutionary changes in functional traits “scale up” to alter the functioning and stability of communities and ecosystems.

Here, we propose that human actions such as assisted dispersal of exotic species to new ranges often result in the mixing and reorganization of genetic variation that can shift the mean and variance of key functional traits that are part of ecological feedback loops, and in so doing trigger a critical transition within invaded ecosystems. We first discuss the genetic and ecological processes at work during invasion that may produce changes in functional traits within invasive populations, and proceed by discussing how these changes in functional traits can alter ecological feedbacks that have community and ecosystem-level consequences. We then illustrate how a theoretical modeling framework can be used to predict how genotypic variation in functional traits may scale up to have community and ecosystem consequences, and provide a specific case study using the invasive grass, *Phalaris arundinacea,* an aggressive invader of North American wetlands (Lavergne and Molofsky 2004). We conclude by exploring how experimental approaches can contribute to a more detailed understanding of how artificially increasing admixture between ecologically and evolutionary distinct populations via human-aided dispersal can promote ecosystem transformation.

## Genetic Changes in Functional Traits of Invasive Species as Drivers of Ecosystem Transformation

Invasive species can reveal novel insights into how genetic changes that affect functional trait variation within a species can lead to changes in community diversity and ecosystem-level reorganization. This is because both the ecological and genetic contexts under which populations evolve are subject to change during invasion, and hence invasions represent unintended and often sudden perturbations to the evolutionary trajectory of populations and their interactions with other components of the ecosystem that can be studied and quantified (Sax et al. 2005).

For years, the study of invasive species has focused on testing specific hypotheses about the ecological mechanisms leading to dominance of introduced invasive species (e.g., enemy release hypothesis, novel weapons hypothesis, and the accumulation of local pathogens hypothesis) (Callaway and Ridenour 2004; Levine et al. 2004; Wolfe et al. 2004; Eppinga et al. 2006). Yet invasions also profoundly alter the genetic structure of introduced populations and many invasions produce “melting pots” of high genetic diversity that may be linked to invasiveness (Ellstrand and Schierenbeck 2000; Facon et al. 2008; Keller and Taylor 2010). Genetic changes at the genotypic and population level can rapidly shift the mean and variance of functional phenotypic traits, some of which may have ecosystem-level consequences (Eppinga et al. 2011; Eppinga and Molofsky 2013). In such cases, evolutionary changes to functional traits in the invader can cause a cascade of biotic and abiotic changes, ranging from competitive displacement of co-occurring native species to alterations in ecosystem processes, and may even initiate a critical transition in ecosystem state (Van Nes and Scheffer 2004). Thus, genetic changes within invasive populations, through their effects on functional traits, may alter ecological feedbacks and lead to a critical transition from a native-dominated to an invasive-dominated ecosystem.

Admixture is potentially a widespread mechanism for producing phenotypic changes in the invader capable of leading to ecosystem shifts. The mixing and mating between genetically divergent introduction sources can frequently occur during the invasion process when the invasive species has multiple introduction pathways and high propagule pressure (Culley and Hardiman 2009). This may expand the overall genetic and phenotypic variance within introduced populations (Kolbe et al. 2004, 2007, which can lead to an immediate fitness advantage due to hybrid vigor (e.g., Facon et al. 2008; Keller and Taylor 2010) and an enhanced response to selection (Lavergne and Molofsky 2007; Keller et al. 2009; Colautti et al. 2012). Further, by combining diversity from different native range sources, admixture can also produce novel invasive genotypes with new trait values, or even new multi-trait combinations (Calsbeek et al. 2011), including individuals with extreme trait values that exceed either parental source (i.e., transgressive segregation sensu Rieseberg et al. 1999, 2007). Moreover, evidence now exists that invasions can be accompanied by dramatic changes in the overall size and structure of the genome itself, with apparent associations between genome size and functional traits that influence ecosystem and community properties (Grotkopp et al. 2004; Kolbe et al. 2007; Lavergne et al. 2010b; Bainard et al. 2012).

What are the consequences for native communities and ecosystems when an invader has expanded functional trait variation and competes for similar niche space with native species? How might admixture and its effects on functional traits mechanistically act to alter both the outcome of competition and ecosystem function? Studies have documented that invasive species are capable of substantially altering native ecosystems, including changes to nutrient cycling, litter dynamics, and hydrology (Vitousek et al. 1987; Witkowski 1991; Ehrenfeld 2003, 2010; Fischer et al. 2010). One functional change that is commonly found in invasive populations when compared to their native range counterparts is that invasive plants are larger in the new range. This could arise due to hybrid vigor (e.g., heterosis) following admixture, or because the release of plants from specialist herbivores found in the native range allows for selection for reallocation of resources from defense into growth (Blossey and Nötzold^1995^; Bossdorf et al. 2005). In fact, increased biomass production in invasive populations has been demonstrated for many invasive species (Blumenthal and Hufbauer 2007), and may be a key trait capable of altering ecosystem processes if larger plants leave more litter (i.e., undecomposed biomass) within the ecosystem, which can shift community composition through ecological feedback loops involving light and soil nutrients (Eppinga et al. 2011; Eppinga and Molofsky 2013; Kaproth, Eppinga and Molofsky 2013). Perhaps the best-documented example involves Chinese Tallow tree, where increased growth capabilities and greater biomass production have evolved during its invasion in North America, leading to profound ecosystem effects (Siemann and Rogers 2001; Zou et al. 2006, 2008). In addition, changes in chemical composition (leaf C:N ratio) or physical characteristics (amount of lignin in the leaf) could alter litter decomposition rates, leading to alteration in feedback loops between native and invasive plants (Zhou et al. 2008). At the species level, a recent phylogenetically controlled experimental study comparing leaf-litter decomposition rates between native and invasive plant species found that leaf litter from invasive species has a slower rate of decomposition than leaf litter from native species (Godoy et al. 2010). At the intraspecific level, genetically based differences in leaf characteristics have been found to alter litter decomposition in at least three invasive tree species (Zou et al. 2006; Silfver et al. 2007; Lecerf and Chauvet 2008). Another ecosystem change induced by genetic changes in functional traits of plant invaders is the alteration of communities of insect herbivores, soil microbes, and soil invertebrates (Neira et al. 2005; Karley et al. 2008; Lankau 2011).

The evidence from these studies on invasive plants suggests that changes in the population variance for key traits such as greater biomass production, or more recalcitrant litter, may lead to a tipping point where the ecological dynamics such as species interactions or ecosystem processes shift, creating a critical transition that favors the establishment and dominance of the invader over the native biological community (Scheffer 2009; Eppinga et al. 2011). Once critical transitions occur, they can result in a cascade of effects on ecosystem functions that can be difficult to reverse (Crooks 2002; Cuddington and Hastings 2004; Cuddington et al. 2007).

## A Theoretical Model Framework Linking Intraspecific Trait Variation to Invaded Ecosystems

**Figure 1:**
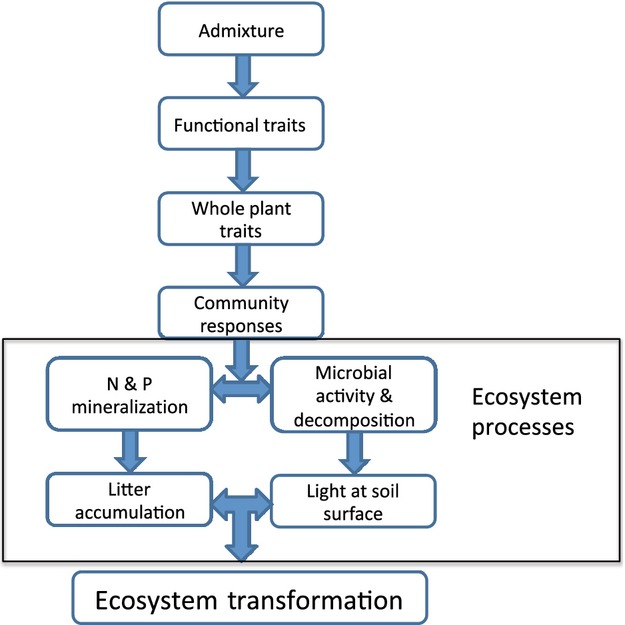
Genes to ecosystem cascade initiated by admixture and culminating in community and ecosystem-scale transitions in structure and function.

To understand the cascade of changes between intraspecific variation in the invader's traits to native community diversity and ecosystem function, we need to first identify the feedback mechanisms that can result in an ecosystem transformation and then understand how these feedback relationships are altered by admixture creating expanded functional trait variation. Theoretical models can allow us to determine when the proposed feedback mechanisms are large enough to cause a critical transition; models can also point to important follow-up experimental studies that focus on determining whether a feedback mechanism of sufficient magnitude is likely to be achieved in natural settings and under what environmental conditions (see Eppinga and Molofsky 2013 for an example). For example, controlled crosses that recreate the effects of admixture between invasion sources could be coupled with experimental field studies that allow us to better understand the relationship between trait expression in the invader and community and ecosystem responses (Fig. 1). Such studies are possible in invasive plant species in which important feedbacks leading to an ecosystem change have been identified and for which genotypes from known invasion sources from the native range can be experimentally hybridized.

Below, we develop a theoretical framework for understanding how the cascade of changes from admixture through functional trait variation to alteration in community and ecosystem function can lead to an ecosystem transition to a novel ecosystem state (Fig. 1). Our starting point involves a theoretical exploration of how dominant feedback relationships in ecosystems can be altered by changes in species' resource-competition relationships, which we then later use to incorporate the effect of expanded genotypic variation in functional traits on the ecological feedbacks. To this end, we illustrate our approach using a modified version of the classical resource-competition model (Tilman 1982), in which two plant populations compete for a soil nutrient and light (Díaz-Sierra et al. 2010; Eppinga et al. 2011). In this model, plant growth is limited by either soil nutrient availability or light availability:


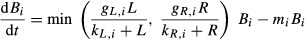
(1)

In which, *B*_i_ indicates the biomass of species *i* (see Table 1 for dimensions of all parameters and state variables), *L* is light availability and *R* is soil resource availability. In addition, *g*_*J,i*_ indicates the maximum growth rate if resource *J* is limiting, *k*_*J,i*_ is the resource density at which plant species *i* reaches half its maximum growth rate, and *m*_*i*_ indicates losses of plant species *i* due to mortality. In turn, dynamics in soil nutrient availability are governed by nutrient supply and plant uptake by co-occurring species in the community (where we distinguish native (N) and invasive (I) species):

**Table 1:** Model parameters and state variables. Parameters are expressed in dimensions of time (T), length (L), Energy (E), Mass of soil resource (M_R_), plant mass (M_S_), and/or litter mass (M_D_).

Symbol	Interpretation	Dimensions
*g*_*L,N*_	Maximum growth rate native population under light limitation	T^−1^
*g*_*L,I*_	Maximum growth rate invasive population under light limitation	T^−1^
*k*_*L,N*_	Light availability at which the native population reaches half its maximal growth rate (if light limited)	EL^−2^
*k*_*L,I*_	Light availability at which the invasive population reaches half its maximal growth rate (if light limited)	EL^−2^
*g*_*R,N*_	Maximum growth rate native population under nutrient limitation	T^−1^
*g*_*R,I*_	Maximum growth rate invasive population under nutrient limitation	T^−1^
*k*_*R,N*_	Nutrient availability at which the native population reaches half its maximal growth rate (if nutrient limited)	M_R_M_S_^−1^
*k*_*R,I*_	Nutrient availability at which the invasive population reaches half its maximal growth rate (if nutrient limited)	M_R_M_S_^−1^
*m*_*N*_	Mortality rate native population	T^−1^
*m*_*I*_	Mortality rate invasive population	T^−1^
*a*	Turnover rate of nutrient supply	T^−1^
*S*	Nutrient availability in absence of both populations	M_R_M_S_^−1^
*q*_*R,N*_	Nutrient content of tissue of native population	M_R_M_P_^−1^
*q*_*R,I*_	Nutrient content of tissue of invasive population	M_R_M_P_^−1^
*ρ*	Soil bulk density	M_S_.L^−3^
*l*_*Root*_	Rooting depth of both populations	L
*Q*_*R,N*_	Nutrient content of native population litter at which it decomposes at rate *d*_*N*_	M_R_M_D_^−1^
*Q*_*R,I*_	Nutrient content of invasive population litter at which it decomposes at rate *d*_*I*_	M_R_M_D_^−1^
*α*_*R,N*_	Nutrient-litter feedback coefficient native population	-
*α*_*R,I*_	Nutrient-litter feedback coefficient invasive population	–
*d*_*N*_	Decomposition rate of native population's litter	T^−1^
*d*_*I*_	Decomposition rate of invasive population's litter	T^−1^
*L*_*0*_	Light supply rate	EL^−2^T^−1^
*γ*_*L,N*_	Light interception coefficient native population	L^2^M_P_^−1^
*γ*_*L,I*_	Light interception coefficient invasive population	L^2^M_P_^−1^
*α*_*L,N*_	Light-litter feedback coefficient native population	L^2^M_D_^−1^
*α*_*L,I*_	Light-litter feedback coefficient invasive population	L^2^M_D_^−1^
*B*_*N*_	Aboveground living biomass of native population	M_p_L^−2^
*B*_*I*_	Aboveground living biomass of invasive population	M_p_L^−2^
*R*	Soil resource availability	M_R_M_S_^−1^
*D*_*N*_	Aboveground litter mass of native population	M_D_L^−2^
*D*_*I*_	Aboveground litter mass of invasive population	M_D_L^−2^
*L*	Light availability	EL^−2^T^−1^
*t*	Time	T


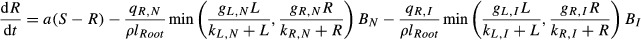
(2)

which *B*_*N*_ indicates native species biomass and *B*_*I*_ invasive species biomass. Further, *a* is the rate at which nutrients are replenished, *S* the soil nutrient availability in the absence of plants, *q*_*R,i*_ the nitrogen content of plant species *i*, and *ρ* the bulk density of the soil and *l*_*Root*_ the plant rooting depth. Plant material intercepts light, meaning that light availability decreases with increasing plant biomass density. Because this process occurs on a much faster timescale than the processes mentioned above, a quasi-steady state can be assumed (Reynolds and Pacala 1993):


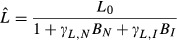
(3)

Previous research suggests that novel, invasive genotypes such as those produced by admixture may undergo a shift in biomass production and carbon:nitrogen ratios in leaf tissue (Blumenthal and Hufbauer 2007; Eppinga et al. 2011). Even without knowing the exact genetic mechanisms that can lead to these trait changes, the potential consequences of such a trait change can be explored by integrating these functional traits into our model framework. More specifically, in the model, the carbon:nitrogen ratio is defined by 1/*q*_*R,i*_. Thus, an increase in the carbon:nitrogen ratio corresponds to a decreasing value of *q*_*R,i*_. Thus, changing this parameter alters the consumption vector of the invasive population (see Appendix S1 in the Supporting Information for the analytical analysis leading to the results presented in this section).

**Figure 2:**
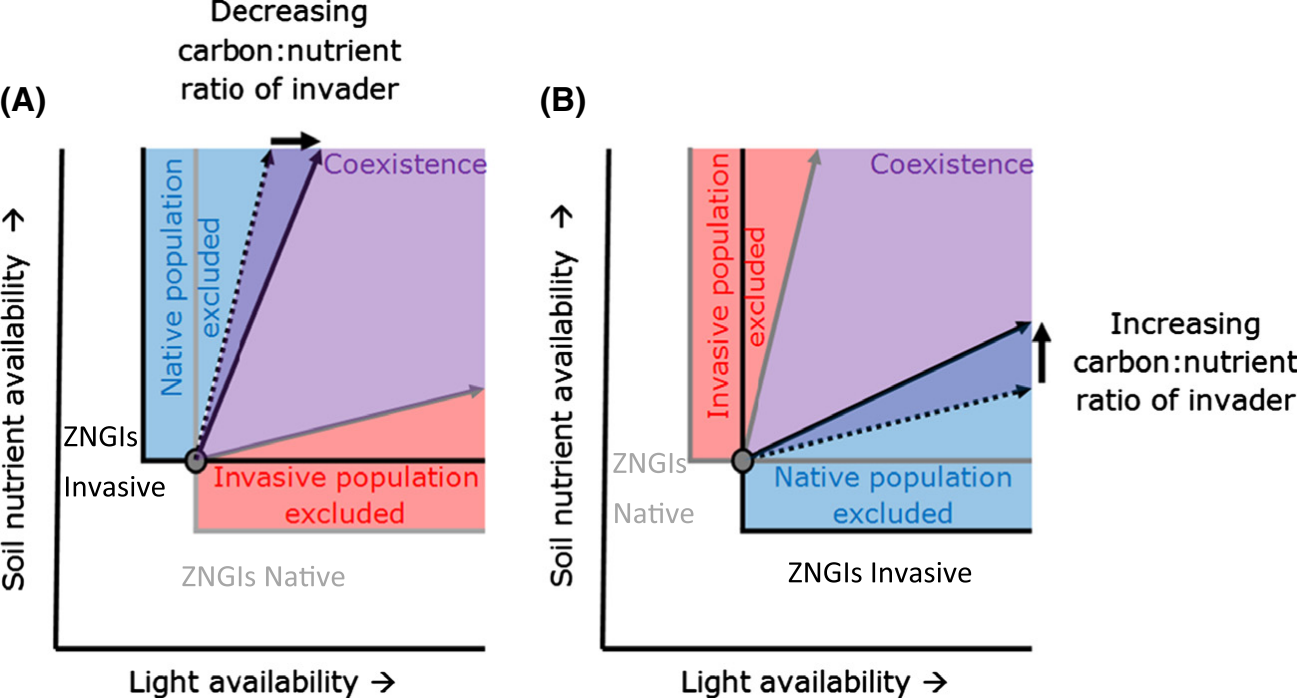
Zero Net Growth Isocline (ZNGI, cf. Tilman 1982) diagrams assessing the effect of an invading population's change in mean trait value when in competition with a native population. Isoclines and consumption vectors of the invasive population are drawn in black, isoclines, and consumption vectors of the native population are drawn in gray. Considering competition for soil nitrogen, a change in an invader's inherent leaf tissue carbon:nitrogen ratio can result in a competitive advantage if: (A) the invader is the stronger competitor for nutrients and decreases its leaf tissue carbon:nitrogen ratio; (B) the invader is the weaker competitor for nutrients and increases its leaf tissue carbon:nitrogen ratio.

Two cases can be distinguished in which a changing trait value may affect the outcome of plant competition (Fig. 2). The first case, often cited as a means to enhance competitive success of invasive populations, is that populations achieve competitive success by attaining a relatively low leaf tissue carbon:nitrogen ratio (Liao et al. 2008; Ehrenfeld 2010). This possibility emerges here in our model framework as well. A decrease in the invader's leaf tissue carbon:nitrogen ratio will increase the slope of the consumption vector (Fig. 2A, cf. Tilman 1982; Appendix S1). This change may increase the invasive population's success, provided that it is a relatively poor competitor for light (Fig. 2A).

In the second case, an increase in the invader's leaf tissue carbon:nitrogen ratio decreases the slope of the consumption vector (Fig. 2B, cf. Tilman 1982; Appendix S1). Figure 2B shows that this increase in leaf C:N may favor the dominance of the invasive population, provided that the invader is a relatively weak competitor for soil nutrients. This is a likely scenario when an invasive species has been introduced without its specific root mycorrhizae (Nuñez et al. 2010), for example.

Until now, the framework does not yet include the possibility of feedbacks between ecosystem characteristics and the response of plant populations. These feedbacks can be included by explicitly incorporating litter dynamics in the model (Daufresne and Hedin 2005; Eppinga et al. 2011):


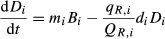
(4)

in which *D*_*i*_ indicates the density of litter produced by species *i*. The decomposition rate of this litter depends on its quality. *Q*_*R,i*_, which indicates the nutrient content of the litter at which it decomposes at rate *d*_*i*_. Litter of lower quality (i.e., smaller values *of q*_*R,i*_) will decompose slower, higher quality litter decomposes faster.

If litter dynamics are included, feedbacks can arise: competitive interactions between the native and invasive plant species then determine the quantity and quality of litter formed, which in turns modifies environmental conditions (i.e., soil nitrogen and light availability), which feeds back to the competitive ability of both species (Daufresne and Hedin 2005; Eppinga et al. 2011). Incorporating these processes in the equations describing soil nitrogen availability (Eppinga et al. 2011) yields:


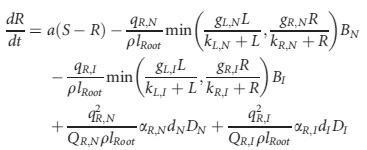
(5)

in which *α*_*R,I*_ indicates the mineralization efficiency of plant species *i*'s litter. As litter decomposition now affects nutrient availability, which in turn will feed back on effects on plant growth, *α*_*R,I*_ can be seen as nutrient-litter feedback coefficients. As noted above, litter will also intercept light, meaning that light availability is now described by:


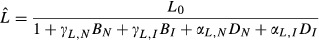
(6)

in which *α*_*L,i*_ indicates the per capita interception of light by litter produced by species *i*. As litter decomposition now affects light availability, which in turn will feed back on effects on plant growth, *α*_*L,i*_ can be seen as light-litter feedback coefficients.

## The Admixture, Trait Variation, Ecological Feedback Loop Leading to Ecosystem Transformation

As argued in the previous section, post-introduction admixture can lead to a quantitative change in the expression of functional traits and, if those traits are heritable, can initiate or amplify ecological feedback effects, potentially inducing irreversible changes in ecosystem structure. Our model highlights a particular case where trait alteration in resource uptake and allocation strategies might induce such a cascading effect on ecosystem functioning. More specifically, due to the potential of litter feedbacks, a change in the invader's leaf tissue carbon:nitrogen ratio can affect the competitive ability of other plant species as well. Thus, ecosystem transformation can now arise when the invader-induced changes in traits (in this case greater litter production) caused by admixture, and possibly also post-admixture phenotypic selection, shifts the trait distribution of the invasive population past the predicted tipping point. The proposed framework can be used to analyze how admixture-induced changes in the mean or variance of key functional traits would affect ecosystem stability.

**Figure 3:**
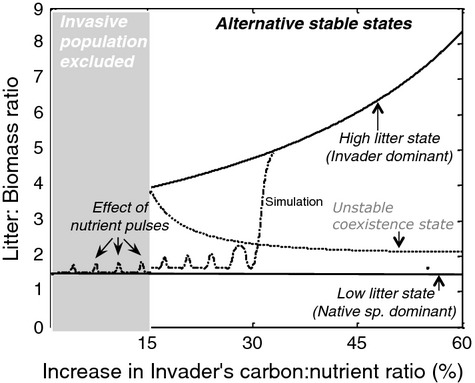
Litter feedbacks and evolutionary change toward a higher carbon:nitrogen ratio synergistically change the outcome of competition between native and invasive populations. In habitats where the invasive is not present yet, the invasion process may encompass a critical transition from a low-litter state to a higher litter state but to do so may require a disturbance. This is modeled in the figure as nutrient pulses (as could occur following a soil disturbance, or episodic runoff). The *y* axis of the graph illustrates the change in the litter:biomass ratio following these nutrient pulses. Nutrient pulses were modeled as a short-term (100 days) increase in nutrient supply (up to S = 100 mg·kg^−1^) and the carbon:nitrogen ratio of the invader's leaf tissue increases during the simulation. Thus, the effect of nutrient pulses becomes more pronounced, eventually forcing the system over a threshold in the litter: biomass ratio, coinciding with a critical transition toward a high-litter, invader-dominated state. The dotted line indicates the litter:biomass ratio of the (unstable) coexistence equilibrium. This dotted line is not a formal separatrix.

Applying the above framework to the invasion of *Phalaris arundinacea* in North American wetlands, Eppinga et al. (2011) showed that in ecosystems where the original trait value of the invader would not enable invasion, a phenotypic change toward higher carbon:nitrogen ratios would enable a critical transition toward an invader-dominated state when the invader's leaf tissue carbon:nitrogen ratio passes a critical threshold (Eppinga et al. 2011; Fig. 3). An indicator for the litter conditions favoring *Phalaris* dominance is the ratio between the amount of litter present and the living aboveground biomass because invasive *Phalaris* are better competitors for light but poorer competitors for nutrients, so they have a competitive advantage when light is more limiting; and greater litter results in an overall greater amount of nutrients present in the system over a longer time period. In addition, the higher the C:N ratio present in the system will decompose more slowly, resulting in a higher overall litter:above-ground biomass ratio. Model simulations suggest that a critical transition to a high-litter to biomass state can be triggered by an ecological disturbance such as pulses in available nutrients arising from eutrophication, for instance (Fig. 3). As *Phalaris* is a strong competitor for light, it benefits from high-litter conditions. An indicator for the litter conditions favoring *Phalaris* dominance is the ratio between the amount of litter present and the living aboveground biomass. This litter:biomass ratio is likely to increase when the proportion of invasive (i.e., high leaf tissue C:N) *Phalaris* genotypes in the system increases. This can initiate a positive litter feedback, as reduced competition from native species may outweigh the negative effect of litter accumulation on the growth of invasive *Phalaris* genotypes. Model analyses suggest that this positive litter feedback may be initiated above a certain threshold in the litter:biomass ratio (Fig. 3; Supporting Information).

Our previous work (Lavergne and Molofsky 2007) focused on the effect of admixture on mean trait values in invasive populations. Such shifts in trait means can be established by admixture and repeated episodes of directional selection. To obtain a mechanistic understanding of selection requires explicit consideration of additive genetic variation within populations and selection among invasive genotypes in the new habitat. However, depending on the amount of additive genetic variance and the strength of selection, this process can be gradual and require multiple generations to shift the mean far enough to elicit ecosystem transformation.

In the previous sections, we discussed the role that admixture during invasion plays in the rapid emergence of novel genotypes with trait values that differ from the parental types, and which can produce large phenotypic variance in functional traits at a faster rate than would occur through mutation and selection, often in one or two generations (Lynch 1991; Lynch and Walsh 1998). Now, instead of examining the *mean* trait of invasive genotypes, we can examine the conditions that lead to the dominance of an invasive plant species by explicitly considering the *variance* between genotypes that is commonly increased by admixture among divergent sources during invasion. The development of this type of genotypically diverse invasive population can be described by reformulating the above framework into a spatially explicit, stochastic, individual-based model (e.g., Eppstein and Molofsky 2007), which considers the fitness of different invasive genotypes competing with each other (through intraspecific competition leading to selection) and with a native species (through interspecific competition). In this model, we create a lattice of cells, in which each cell can be occupied by one individual: a native species, or a particular genotype of the invasive species. After individual growth during the growing season, as described by the non-spatially explicit equations above, the probability of a genotype *i* occupying a cell the next season is determined by a transition probability, which depends on the per capita potential growth rate of genotype *i*, relative to the growth rate of all competitors in the nearby cells comprising the interaction neighborhood (Eppstein and Molofsky 2007; Eppinga et al. 2013):


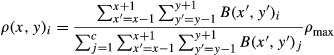
(7)

in which *ρ*(*x*,*y*)_*i*_ is the probability of occupation of the cell at position (x,y) by genotype *i* in the next timestep, *C* is the total number of genotypes and species in the community. Further, the summations over *x′* and *y′* indicate the size of the assumed interaction neighborhood (here we use a discrete neighborhood (Moore)). By including genotypes that vary in their C:N ratio in the simulations as is expected post-admixture, we can investigate how genetically determined variation in this key trait (leaf C:N ratio) affects the potential spatial spread of *Phalaris* and triggers a transition.

We parameterize the model to reflect the invasion of North American wetlands by introduced European genotypes of reed canarygrass, *Phalaris arundinacea*. Therefore, we simulate competition between a phenotypically diverse *Phalaris* population and a native species along a nutrient supply gradient. To parameterize the model, we include 10 genotypes spanning the range of C:N ratios observed in natural populations of invasive *Phalaris* that are the products of admixture between divergent native range sources (Lavergne and Molofsky 2007; Eppinga et al. 2011; Eppinga and Molofsky 2013). To focus on the effect of variation in tissue C:N, we keep other differences between genotypes constant. We note that while this approach does not explicitly model the quantitative genetic processes that produce standing genotypic variation (e.g., mating, recombination, and segregation), it does effectively model how the spatial spread dynamics change in the presence of genotypic variation in functional traits, which is our key objective. Such genotypic variation can easily arise in one or a few generations, following admixture of divergent sources (Lynch 1991), and does not require mutation per se, which makes it particularly applicable to biological invasions (Ellstrand and Schierenbeck 2000; Kolbe et al. 2004; Dlugosch and Parker 2008). Further, many invaders (notably many plant species) are capable of strong asexual reproduction, making it highly plausible that once a particularly invasive genotype arises, it may persist and spread without recombination breaking up its beneficial gene and trait combinations.

**Figure 4:**
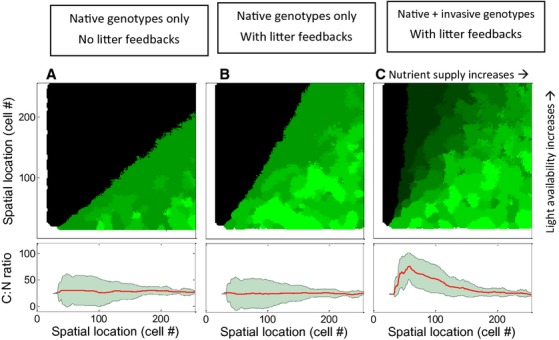
Stochastic cellular automata simulations of competitive dynamics between a native wetland species (in black) and an invasive population of multiple genotypes that differ in leaf tissue C:N ratio (in shades of green). We modelled five native *Phalaris* genotypes with C:N ratios ranging between 20 and 30 g_C_·g_N_^−1^, and five invasive genotypes with C:N ratios ranging between 35 and 90 g_C_·g_N_^−1^. Model simulations were run over 150 seasons for 150 days, considered a range of soil resource input (increasing from 0 to 30 mg·kg^−1^) and light availability (increasing from 0 to 50 mol·m^−2^·day^−1^). (A) If we consider only *Phalaris* native genotypes (black), we find that the genotypes can only invade nutrient-rich areas with relatively low light availability; (B) Here, we consider only *Phalaris* native genotypes but include the dynamics of a litter feedback, (C) Invasive success increases as novel genotypes with specific C:N traits are included, the invasive genotypes dominate the *Phalaris* population at low nutrient supply regimes, which is reflected by a higher average C:N ratio within the population.

Our model results show that litter feedbacks may increase the success of *Phalaris* in competition with native species; certain invasive genotypes are able to invade more oligotrophic, nutrient poor wetlands than was possible without incorporating genotypic variation (Fig. 4). Further, the results show that by incorporating the expanded phenotypic variation as observed in admixed invasive populations, we include the presence of high C:N genotypes which increases the range of conditions where the population becomes invasive. Interestingly, when there is phenotypic variation in *Phalaris*' C:N ratio, without assuming any other tradeoff, we find that particular genotypes with higher inherent C:N ratio are able to expand *Phalaris'* range into previously unoccupied environmental niche regions (Fig. 4).

Although trait variation can be measured straightforwardly in greenhouse or common garden experiments, it is not always clear how variation in traits measured under controlled environmental conditions affects ecosystem processes, and possible feedbacks therein. Analytical approaches, such as the framework presented here, provide an opportunity to scale up the effects of phenotypic variation observed in controlled environment experiments to the ecosystem level. Interestingly, the results of model analyses can lead to predictions of how the occurrence of increased phenotypic variance due to human-aided dispersal and admixture is reflected in the characteristics of the ecosystems invaded. In the presented example, litter feedbacks induced by increases in the invader's leaf tissue carbon:nitrogen ratio should be reflected by higher litter:biomass ratios in invaded ecosystems. Higher leaf C:N has indeed been observed for invasive *Phalaris arundinacea*, confirming a central prediction of the modeling approach described here (Eppinga and Molofsky 2013).

## Conclusions and Prospects for Future Work

**Figure 5:**
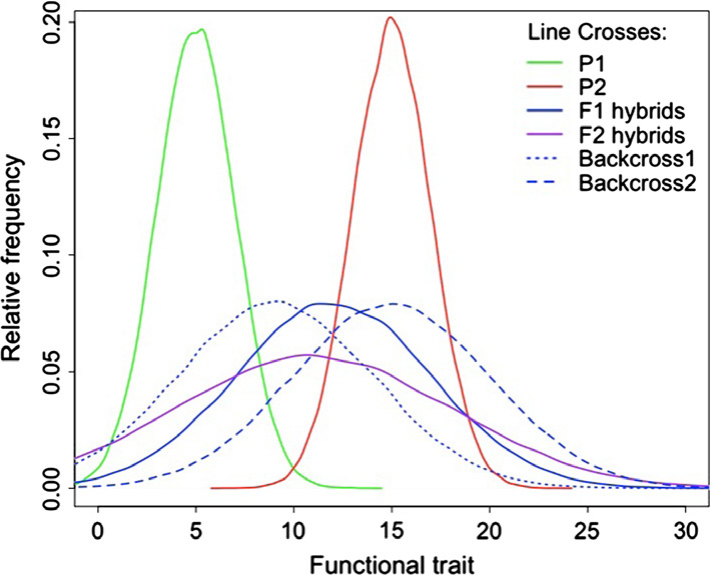
Theoretical prediction of functional trait distributions from line crosses between divergent “parental” populations (P1 and P2) for early- and advanced hybrid generations (F1, F2 and backcrosses). Note the expanded phenotypic variance in hybrids reflecting the production of transgressive genotypes segregating trait values beyond the range of either parental population.

The occurrence of expanded functional trait values or new strategies of growth or resource capture following admixture may be one mechanism whereby invasive genotypes develop the ability to transform ecosystems. The effect of admixture on ecosystem transformation can be tested experimentally by creating hybrid genotypes between different invasion sources and testing whether the experimentally admixed population possesses expanded trait variation or multi-trait combinations. First one must test the consequence of admixture for variation in functional traits, which can be done with most species amenable to crossing and analyzed using the quantitative genetic method of line crosses (Lynch and Walsh 1998; Fig. 5). This represents a powerful manipulative method to evaluate how the admixture of divergent native range lineages impacts the distribution of key traits in experimental hybrid genotypes, and how these traits compare to current invasive genotypes. Trait-based research is an increasing focus area in community and ecosystem ecology (McGill et al. 2006; Albert et al. 2010), but to date studies on intraspecific variation in traits and how trait variation can alter feedbacks and influence ecosystem dynamics has been under-explored. Trait variation in invasive populations represents an important subset of trait-based research and can provide important information about the processes generating variation in traits and multi-trait combinations, and their effects on ecosystems.

Obviously, not all changes in traits will cause an ecosystem transformation. Therefore, it is important to not only document changes in trait expression post-admixture, but also experimentally determine whether the new trait values found in hybrid genotypes are sufficiently different than trait values in the parental populations so as to induce a critical transition. While our resource-competition model can help identify the necessary conditions in intraspecific trait variation necessary for a critical transition, experimental tests in situ still provide the gold standard for understanding whether expanded trait variation is altering feedback relationships and ecosystem state. Such studies are possible in ecosystems consisting of perennial or annual plants that can be easily manipulated and studied in mesocosm experiments. Thus, plants with known genetic backgrounds (e.g., parentals or hybrids of varying generations) can be planted into plots with the idea that the resulting ecosystem properties can be tested pre- and post-introduction. Such data would provide a mechanistic understanding of the role that genotypic variation and even genotype-specific traits in an invasive population have on ecosystem properties. While these experiments would no doubt be challenging to conduct, requiring many replicate gardens or mesocosms to capture the effects of genotypic variation, they are feasible, especially for plants that can be clonally propagated for experimentation. Further, while these types of experiments would likely involve a complex series of predictors and hypothesized feedback relationships, the relationships could be synthesized using tools such as structural equation modeling (SEM) that combines path analysis and factor analysis to compare specific hypotheses about how different variables in the system interrelate (Grace 2006).

We have outlined a synthetic framework that incorporates model predictions and experimental approaches to determining how variance in trait expression during invasion may lead to ecosystem transformation. Our study has indicated that the enhanced trait variation found in introduced populations of invasive species brought about through admixture can ultimately alter the diversity and structure of natural communities, reduce ecosystem resilience, and ultimately be responsible for abrupt shifts in ecosystem states. Moreover, studying the extreme trait variance that results from admixed genotypes post-introduction can enhance our understanding of how intraspecific diversity in functional traits can alter ecosystem function and ultimately lead to a better understanding of how the combined effects of both ecological and evolutionary dynamics interact in natural populations. A comprehensive approach that combines theoretical modeling, quantitative genetics, and manipulative field studies will lead to a deeper understanding of how human-aided dispersal of populations to new ranges can be a key step in the ecological and evolutionary processes that lead to critical transitions to a new ecosystem state.

## Supporting Information

Appendix S1 Analytical justification of the model results.
