# Supplementary material for: Human-aided admixture may fuel ecosystem transformation during biological invasions: theoretical and experimental evidence
Source: Ecol Evol. 2014 Feb 23;4(7):899–910. doi: 10.1002/ece3.966 (PMC3997308; doi:10.1002/ece3.966)
Supplement: Appendix S1 — Analytical justification of the model results. [file ece30004-0899-sd1.doc]

Supporting Information: Appendix S1

Here we provide the analytical justification of the results presented in the main text. Also, we show numerical simulations explaining the shapes of the graphs shows in the conceptual Fig. 3 of the manuscript.

As noted in the main text, previous research suggests that novel, invasive genotypes may have undergone an evolutionary shift in expressed carbon:nutrient ratios in leaf tissue (Eppinga *et al.* 2011). This trait will affect the consumption vector of the invasive population, which is expressed as (Eppinga *et al*. 2011):


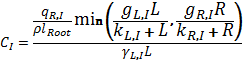
 (S1)

In which *q_R,I_* indicates the nutrient content of the invader’s tissue, and thus inversely related to the invader’s carbon:nutrient ratio. From equation S1 it follows that a higher carbon:nutrient ratio (i.e. a decreasing value of *q_R,I_*) decreases the slope of the invader’s consumption vector, whereas a lower carbon:nutrient ratio (i.e. an increasing value of *q_R,I_*) increases the slope of the invader’s consumption vector.

Using the same model framework as presented in the main text, Eppinga *et al.* (2011) derived the conditions under which an increase in an invader’s leaf tissue carbon:nutrient ratio may alter the outcome of competition with a native population. More specifically, we focused on a case where the system shifted from a state where the invasive population would be excluded into a situation where alternative stable states are possible (as shown in Fig. 3 of the main text). From a quantitative perspective, such a shift would require the slope of the invader’s nutrient-light consumption ratio to become smaller than the nutrient-light supply ratio in the system. Eppinga *et al*. (2011) showed that this occurs when:


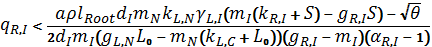
 (S4)

In which:


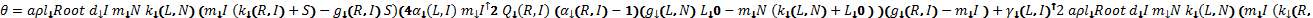
 (S5)

The following parameter values were assigned in Fig. 3 of the main text (following Eppinga *et al.* 2011):

*g_L,N_*= 0.25 day^-1^, *g_L,I_*=0.25 day^-1^, *k_L,N_*= 50 mol.m^-2^.day^-1^, *k_L,I_*= 35 mol.m^-2^.day^-1^, *g_R,N_*= 0.25 day^-1^,*g_R,I_*= 0.25 day^-1^, *k_R,N_*= 30 mg.kg^-1^, *k_R,I_*= 35 mg.kg^-1^, *m_N_*=0.005 day^-1^, *m_I_*=0.01 day^-1^, *a*= 0.005 day^-1^, *q_R,N_*= 15 mg.g^-1^, *q_R,I_*= 15 mg.g^-1^, *ρ*= 530 g.m^-3^, *l_Root_*= 1 m, *Q_R,N_*= 15 mg.g^-1^, *Q_R,I_*= 15 mg.g^-1^,*α_R,N_*= 0.7, *α_R,I_*= 0.7, *d_N_*= 0.003 day^-1^, *d_I_*= 0.003 day^-1^, *L_0_*= 50 mol.m^-2^.day^-1^, *γ_L,N_*= 0.03 m^2^.g^-1^, *γ_L,I_*= 0.04 m^2^.g^-1^, *α_R,N_*= 0.01 m^2^.g^-1^, *α_R,I_*= 0.013 m^2^.g^-1^, *S*=8 mg.kg^-1^. The same parameterization is used in Fig. 4 of the main text, except for the parameters *L_0_, S* and *q_R,I_*., as indicated in the figure legend.
